# A liver fibrosis cocktail? Psoriasis, methotrexate and genetic hemochromatosis

**DOI:** 10.1186/1471-5945-5-12

**Published:** 2005-11-29

**Authors:** Joseph Mathew, May Y Leong, Nick Morley, Alastair D Burt

**Affiliations:** 1 Department of Histopathology, Royal Cornwall Hospital, Truro, TR1 3 LJ, UK; 2 Research and Development Unit, Royal Cornwall Hospital, Truro, TR1 3 LJ, UK; 3 School of Clinical and Laboratory Sciences, University of Newcastle upon Tyne, NE2 4HH, UK

## Abstract

**Background:**

Pathologists are often faced with the dilemma of whether to recommend continuation of methotrexate therapy for psoriasis within the context of an existing pro-fibrogenic risk factor, in this instance, patients with genetic hemochromatosis.

**Case presentations:**

We describe our experience with two male psoriatic patients (A and B) on long term methotrexate therapy (cumulative dose A = 1.56 gms and B = 7.88 gms) with hetero- (A) and homozygous (B) genetic hemochromatosis. These patients liver function were monitored with routine biochemical profiling; apart from mild perivenular fibrosis in one patient (B), significant liver fibrosis was not identified in either patient with multiple interval percutaneous liver biopsies; in the latter instance this patient (B) had an additional risk factor of partiality to alcohol.

**Conclusion:**

We conclude that methotrexate therapy is relatively safe in patients with genetic hemochromatosis, with no other risk factor, but caution that the risk of fibrosis be monitored, preferably by non-invasive techniques, or by liver biopsy.

## Background

Liver fibrosis, increased liver collagen content, [1-3] damage to the canals of Hering, [4] and cirrhosis, have been reported in patients being treated with methotrexate for psoriasis [5] or rheumatoid arthritis [6] but not in the context of inflammatory bowel disease;[7,8] some studies suggest that this risk of liver fibrosis is overstated [9-11]. The duration of methotrexate therapy,[12] patient age, weekly oral dosage schedule,[13] cumulative methotrexate dose,[14] risk factors for non-alcoholic steatohepatitis (obesity, diabetes mellitus, glucose intolerance)[6,15-17] and concurrent alcohol ingestion [14,18] have been identified as significant risk factors in the evolution of liver fibrosis in these patients. By contrast long-term low-dose methotrexate therapy, in the absence of excess alcohol ingestion or other profibrogenic agents or diseases, have been reported as being relatively free of the risk of developing fibrosis [19,20]. It has been recommended that baseline pre-treatment liver biopsies are necessary to define the initial histologic status of the liver [21,22]. Although annual, or other suitable interval, liver biopsies are recommended with this treatment, to monitor development of liver fibrosis,[12,13,17,21] its necessity in the first five years of treatment[12] or the need at all for this invasive procedure [11] has been questioned; recent guidelines from the British Society of Gastroenterology do not include methotrexate as an indication for liver biopsy [23]. Ultrasound liver examination [24] and measurement of serum type III procollagen aminopeptide (PIIINP) [25-27] have been suggested as alternatives to liver biopsy; the former is useful only if the US appearances are normal as it is not discriminatory in identifying fibrosis [24]. We reflect on our experience with two patients receiving methotrexate for psoriasis, with genetic hemochromatosis (GH) as an "additional" risk factor for the development of liver fibrosis.

## Case presentations

The clinical histories of two patients (patient A and patient B) with psoriasis, being treated with methotrexate, are summarised. During the course of their treatment, liver biopsies were performed as baseline to rule out and subsequently monitor the development of liver fibrosis as, at the present time PIIINP monitoring of liver fibrois is not available in our institution. The possibility of GH was queried on histological grounds and subsequently confirmed with genotyping.

### Patient A

A 47 year-old male patient presented to the dermatologist October 1998 following an episode of severe flare up of psoriasis, which he had had for 15 years. His condition improved with coal tar, hydrocortisone ointment and UVB. However, after stopping UVB, his flare up recurred. Methotrexate was introduced to his treatment regime. After a satisfactory baseline blood test and a trial dose of 2.5 mg, he started methotrexate (10 mg/week) in April 1999. Two weeks later the dose was increased to 15 mg/week. In June 1999, the dose was increased to 17.5 mg/week and then back to 15 mg/week in July 1999 with improvement of his psoriasis. There was no history of excess alcohol ingestion.

**Figure 1:**
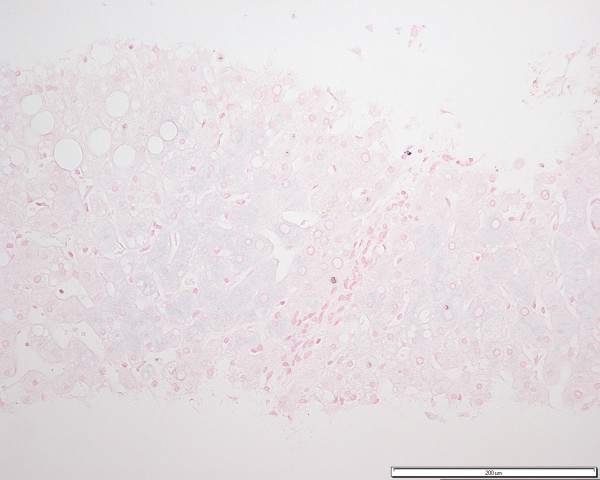
Perl's Prussian Blue stain for iron shows a typical pattern of iron accumulation seen in heterozygous GH (Patient A) (x20)

He had his first liver biopsy, six months later (October 1999) which showed moderate acinar zone 3 and 2 macrovesicular steatosis, with lesser amounts of microvesicular steatosis. Acinar zone 1, grade 1 parenchymal iron accumulation, with an acinar gradient, suggestive of heterozygous form of GH was seen with Perl's Prussian Blue (figure 1); iron was not detected in sinusoidal Kupffer cells or macrophages. There was no evidence of fibrosis or, accumulation of copper or alpha 1 anti-trypsin. Genotypic studies confirmed heterozygosity for the HFE gene mutation H63D (in the absence of C282Y).

He had his second liver biopsy in November 2001; total cumulative methotrexate dose of 1.56 gm from his first liver biopsy. The biopsy showed moderate macrovesicular steatosis, mild steatohepatitis, grade 1 iron accumulation similar to his first biopsy but no fibrosis or contra-indication to the continued use of methotrexate.

### Patient B

This 58 year-old patient was first seen by the dermatologist in October 1992 with psoriasis. He responded well with UVL and Alphosyl HC. In February 1993, his psoriasis got worse and methotrexate was started with a test dose of 5mg. Following satisfactory baseline blood tests, that was gradually increased to 30 mg/week. His skin condition improved significantly with treatment.

He stopped methotrexate before Christmas 1994 but subsequently he restarted it in March 1995. He had a liver biopsy in August 1995 (cumulative methotrexate dose of 2115 mg) which showed moderate steatosis, moderate deposition of hemosiderin within hepatocytes and no evidence of fibrosis. His liver function tests and full blood counts were normal at that time.

**Figure 2:**
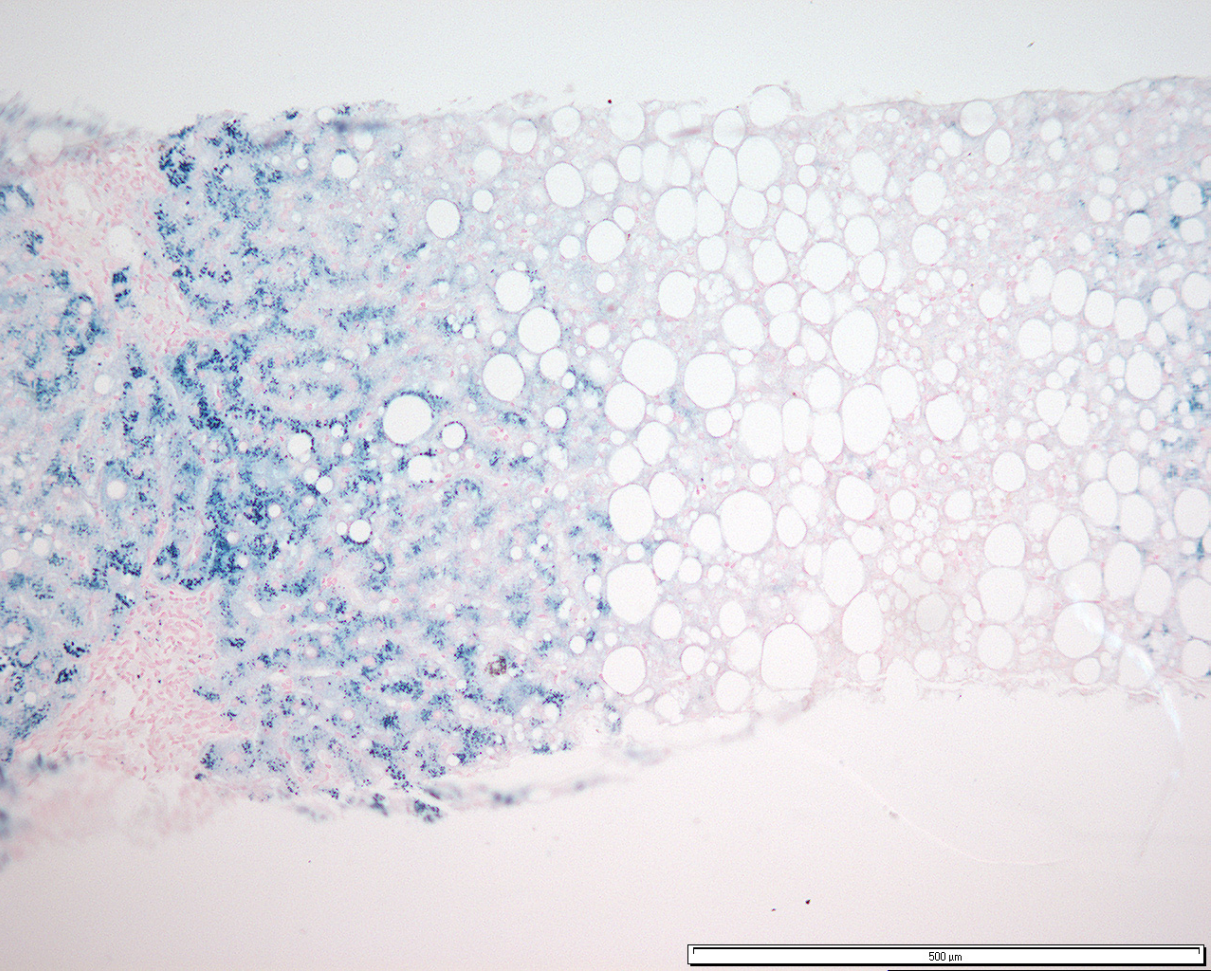
Grade 3 hepatocyte iron accumulation with an acinar distribution pattern consistent with homozygous GH (Patient B) (Perl's Prussian Blue: ×10).

He was lost for follow up for 2 years between May 1996 to May 1998. When he was seen again in May 1998 he had slowly rising ALT and was still on methotrexate. Another liver biopsy (cumulative methotrexate dose of 5000 mg) was performed which showed moderate to severe macrovesicular steatosis with smaller amounts of microvesicular steatosis, predominantly in acinar zones 3 and 2. Grade 3 parenchymal iron accumulation, with an acinar gradient, was seen in acinar zone 1 (Figure 2) suggestive of homozygous GH. There was no evidence of fibrosis and thus no contra-indication for continued use of methotrexate. Genotypic studies confirmed homozygosity for HFE gene mutation C282Y, following which regular venesection was arranged. He continued on methotrexate until March 1999 when his ALT was raised at 96. However, as stopping methotrexate did not improve his liver function test result it was restarted together with UVL therapy in May 1999 for flare up.

**Figure 3:**
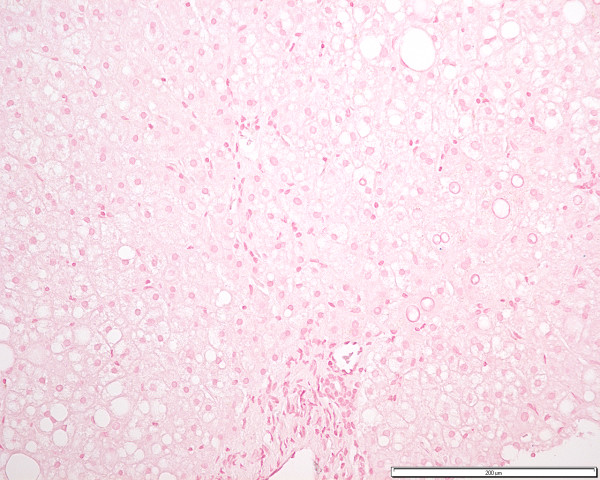
Following venesection (Patient B), there is no histological evidence of residual iron (×20).

**Figure 4:**
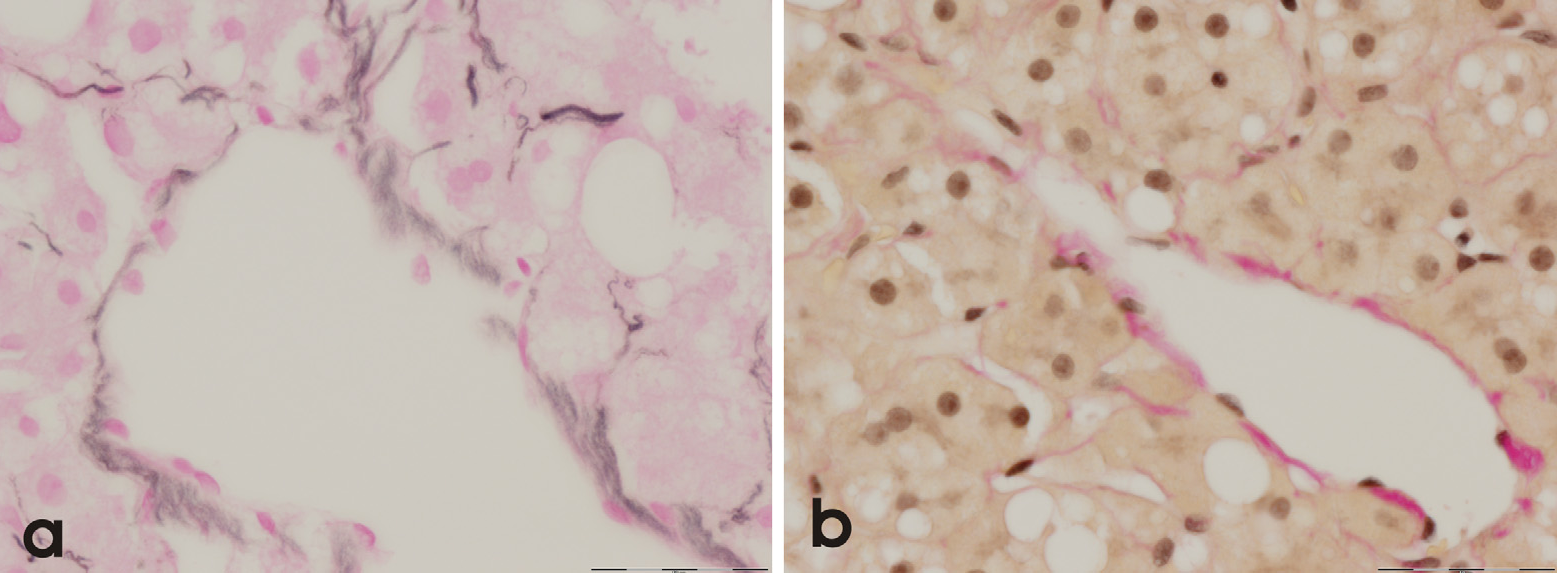
This figure shows a reticulin (a) and a Haematoxylin Van Gieson stain (b) of two terminal hepatic venules in which there is equivocal perivenular fibrosis (x40).

He continued with methotrexate despite failed attempt to reduce the dose. He had another liver biopsy in October 2001 (total cumulative methotrexate dose of 7880 mg) which showed moderate steatosis, moderate to severe steatohepatitis, marginal perivenular fibrosis but no residual iron (Figure 3); the former features were reminiscent of alcohol-induced liver damage (Figure 4). As there was no significant fibrosis, continued methotrexate administration was not contra-indicated although he was advised to curtail his alcohol ingestion.

## Discussion

Pathologists are occasionally faced with a decision of whether to recommend continuation of methotrexate therapy when a patient has an "additional" risk factor for the development of liver fibrosis and/or cirrhosis; in this instance with two patients on long-term methotrexate therapy who had GH. As far as we are aware, there have been no reports in the english literature that addresses this specific issue.

Patients with GH are at risk of developing liver fibrosis and, in the long term, liver cirrhosis; [28-30] there is a also, a risk of developing fibrosis with methotrexate therapy alone [31,32]. Cessation of methotrexate therapy is advised in the presence of significant liver fibrosis [13].

We have described our experience of two hemochromatotic patients on low dose on methotrexate therapy for relatively short periods of three and five years and relatively low cumulative doses. Although one of these patients with homozygous GH was also partial to alcohol, he did not show evidence of significant fibrosis apart from equivocal alcohol-associated perivenular fibrosis. In such instances, adequate counselling of the patient of the risk of excess alcohol in this setting is probably appropriate. There was no evidence of liver damage or fibrosis in our patient with heterozygous GH.

Although measurement of PIIINP can be useful in defining the development of liver fibrosis,[25] elevated or rising levels are not specific or sensitive for liver fibrosis; [25-27] however, liver biopsy has been recommended in patients whose PIINP levels increase from normal whilst they are being monitored for liver fibrosis [27]. Clearly PIIINP measurements will not distinguish fibrosis due to methotrexate or GH, in combination or alone or, fibrosis developing as a result of any other profibrogenic agent. We are unable to offer PIIINP measurements for evaluating liver fibrosis in our institution.

## Conclusion

We conclude that methotrexate therapy is relatively safe in patients with GH but caution that the risk of fibrosis be monitored, preferably by non-invasive techniques, or by liver biopsy. We recommend that patients in this setting be advised of the potential for liver damage especially with "additonal" risk factors.

## List of abbreviations

serum type III procollagen aminopeptide (PIIINP)

genetic hemochromatosis (GH)

ultraviolet B (UVB)

HFE: gene symbol

ultraviolet light (UVL)

alanine aminotransferase (ALT)

## Competing interests

The author(s) declare that they have no competing interests.

## Authors' contributions

JM is the local liver pathologist who made the diagnosis of GH and together with MYL interrogated and prepared the manuscript. ADB provided expert review, advice and contributed to the preparation of the manuscript, as did NM who also initiated contact with these patients and secured consent. All authors have read and approved the manuscript.

## Pre-publication history

The pre-publication history for this paper can be accessed here:


